# Depression after Delivery: Risk Factors, Diagnostic and Therapeutic Considerations

**DOI:** 10.1100/tsw.2007.207

**Published:** 2007-10-22

**Authors:** Debra A. Scrandis, Tehmina M. Sheikh, Robina Niazi, Leonardo H. Tonelli, Teodor T. Postolache

**Affiliations:** ^1^ Mood and Anxiety Program, Department of Psychiatry, University of Maryland School of Medicine, MSTF Building, Room 502, 685 West Baltimore Street, Baltimore, MD 21201, USA; ^2^ School of Nursing, University of Maryland, 655 West Lombard Street, Room 675D, Baltimore MD 21201, USA; ^3^ Residency Training Program, St. Elizabeth's Hospital, 2700 Martin Luther King Avenue, Washington, D.C. 20032, USA

**Keywords:** postpartum depression, postnatal depression, postpartum mood disorders, postpartum blues, depression treatments, women

## Abstract

Postpartum mood disorders can negatively affect women, their offspring, and their families when left untreated. The identification and treatment of postpartum depression remains problematic since health care providers may often not differentiate postpartum blues from depression onset. Recent studies found potentially new risk factors, etiologies, and treatments; thus, possibly improving the untreated postpartum depression rates. This integrated review examined several postpartum psychiatric disorders, postpartum blues, generalized anxiety, obsessive compulsive disorder, post-traumatic stress disorder, and postpartum psychosis for current findings on prevalence, etiologies, risk factors, and postpartum depression treatments.

## INTRODUCTION

Postpartum depression (PPD) is a major public health problem that requires early detection, early intervention, educational efforts to increase awareness, and research efforts to better understand its mechanisms and to find better interventions[1]. Despite the evidence of poor outcomes from chronic depression in women after childbirth, diagnosis and treatment rates for PPD have not shown significant long-term improvement[2]. There are multiple etiologies suggested for these poor detection and management rates. Women may be reluctant to admit to having a mood disorder due to the stigma of mental illness or they may attribute their symptoms, such as mood changes, sleep disturbances, fatigue, and sexual impairment, to the normal postpartum period[3]. Routine screening in primary care provider offices is not being conducted consistently[4], and if it is done, it encounters difficulties of differentiating between PPD and the postpartum blues, which results in inadequate prognostic considerations and treatment options. Once PPD is identified, various therapies are available for treating this disorder. This integrative review examined recent prevalence, etiologies, and risk factors of postpartum psychiatric disorders, as well as diagnostic and treatment options specifically for PPD.

## Postpartum Mood Disorders

Over the years, there has been significant discussion on whether postpartum mood disorders are distinct diagnoses, part of a continuum from mild to severe distress, or the same as major PPD depression at any other point in life[5,6,7,8,9]. More recently, these mood changes, postpartum blues, and PPD depression are thought to be unique disorders with significant symptom differences and comorbidity[10,11].

## Postpartum Blues

Postpartum blues, also known as the baby or maternity blues, is a condition affecting between 20–85% of women after delivery[12,13,14,15]**.** This wide prevalence range results from the use of different instruments to assess tearfulness or sadness, not the complete syndrome of the blues[16]. The postpartum blues is a transient disorder. Symptoms of intermittent tearfulness, despondency, anxiety, and poor concentration usually begin between 3–4 days postpartum and last between 1–7 days[14]. The anxiety symptoms usually relate to situational difficulties, such as breastfeeding problems. Symptoms usually remit within 2 weeks postpartum.

The postpartum blues may be an inappropriate term to describe these symptoms: rather, this time period may be postpartum reactivity, including a heightened labiality of mood in reaction to stimuli resulting possibly from hormonal withdrawal[17].

The neurochemical etiologies of the blues are not conclusive; only two small studies found an association between low serum progesterone (including alloprenanolone) levels at day 2–3 and the maternity blues[18,19]. One animal study found that high levels of 17β-estradiol suppressed 5α-reductase expression and progesterone in the brain, contributing to postpartum mood disorders, particularly the blues[20]. Three recent studies attributed increased degradation of tryptophan to kynurenine (often occurring after immune activation) in the onset of postpartum blues[21,22,23].

The symptoms of the postpartum blues are distinctly different from PPD. During the first 2 weeks postpartum, if women have depressed moods that increase in severity rather than decrease, they may be experiencing PPD. In some high-risk women, the postpartum blues may predict the development of PPD[24].

## Postpartum Depression

PPD is a complex mix of physical, emotional, and behavioral changes that occur after giving birth that are attributed to the genetic, social, and psychological changes associated with having a baby[25]. The Diagnostic and Statistical Manual of Mental Disorders 4$^{th}$ Edition: Text Revised (DSM-IV TR)[26] views PPD as Major Depression with a Postpartum Onset occurring approximately within 4 weeks after delivery. Symptoms include anhedonia, dysphoria, hopelessness, worthlessness, anxiety, inability to sleep while infant is asleep, poor concentration, appetite disturbances, guilt, and suicidal thoughts. Untreated PPD can lead to chronic depression, maternal infant interaction disturbances, suicide, and in rare cases, infanticide[27]. It is important to note that up to 20% of postpartum deaths are attributed to suicide due to PPD[28].

The period prevalence of major PPD is estimated to be 5.7%; however, studies have not found any conclusive evidence for incidence rates[29]. Findings suggested similar epidemiological rates among Latina, African American, and Caucasian women[30,31]. Two separate studies reported PPD prevalence in Native American women[32] and Immigrant Asian Indian women[33]. However, this prevalence rate was based on self-reported PPD symptoms without a specific diagnosis of PPD. Again, limited prevalence data exist on Asian and Pacific postpartum women in the U.K. and New Zealand since rates were self-reported[34,35].

A recent epidemiological study[36] in Denmark examined a register-based cohort of over 2.3 million Danish-born persons to determine the relative risk of becoming a first-time parent (mothers and fathers), and hospital admission or outpatient contact for a mental disorders within 1 year postpartum. Mothers were at highest risk of being admitted for unipolar depressive disorders at 31–60 days postpartum (RR 3.53, 95% CI 2.47–5.05). Primiparous women were at greater risk than multiparous women. Fathers were not significantly at risk for hospital admission or outpatient contact for a mental disorder. This population-based study provided evidence of first-time childbearing and PPD as a significant public health problem that needs quick identification and subsequent rapid treatment[1].

Postpartum anxiety disorders, such as obsessive compulsive disorder (OCD), panic disorder, and post-traumatic stress disorder (PTSD), are common disorders that occur alone or in combination with major PPD. While in certain cases these disorders are closely associated with pregnancy or delivery, in many other cases, it appears that pregnancy and the perinatal period exacerbate previously latent or minimally symptomatic anxiety disorders. The key feature of these disorders is extreme anxiety. In OCD, women have intrusive, repetitive thoughts that may center on harming the infant and they are horrified to have these thoughts. Sometimes, women have certain behaviors that alleviate the anxiety, such as counting or frequently checking locks. Panic disorder is episodic extreme anxiety with physical symptoms, such as shortness of breath and heart palpitations. PTSD has symptoms of recurrent nightmares and reliving past traumatic events, such as sexual abuse[37]. There is limited research on the prevalence of anxiety disorders; however, the rates of OCD and generalized anxiety disorder are higher in the postpartum period than in the general population[38]. One study reported the incidence of PTSD in about 2% of deliveries[39]. One community sample ($n$ = 156) found that 19.8% had at least one anxiety disorder (specifically panic disorder and/or OCD) and depression at 4–7 months postpartum[40], further supporting the comorbidity of these disorders. No studies examining the etiologies for postpartum anxiety disorders were found, and longitudinal studies would be important to identify risk factors and treatments.

## Risk Factors of Postpartum Depression

The strongest predictors of developing PPD include depression and/or anxiety during pregnancy, stressful life events during pregnancy and/or postpartum, low levels of social supports, and personal history of depression prior to pregnancy[24,41]. Studies on family history of depression, anxiety, or PPD show mixed results[42,43,44]. Other risk factors include women with premenstrual dysphoric disorder[45]**;** adolescents, lower income[46] and recent eating disorders[47]. One study ($n$ = 16) provided some support for estrogen/progesterone and PPD onset in women with a previous history of the disorder. These women may be specifically sensitive to the effects of gonadal steroids, thereby contributing to depressive mood[48].

## Diagnostic Challenges of Postpartum Depression

Early detection through screening and early management of PPD are critical in order to decrease poor outcomes for women and their families[49]. Prenatal depression is a high risk factor for the development of PPD, and it is debatable whether PPD may be a continuation of prenatal depression[50]. However, treating prenatal depression was not highly endorsed due to the possible negative effects of medications on the fetus. For instance, case reports suggested that serotonin-selective receptor inhibitors during the third trimester led to newborn withdrawal syndrome[51]. On the other hand, in the past 5–7 years, research on the devastating effects of untreated prenatal depression, such as poor self-care and prenatal care attendance, added to the controversy of antidepressant therapy during pregnancy[52]. Despite these reports, treatment for prenatal depression was reported in only 33% of women diagnosed with depression and who had a previous history of depression ($n$ = 276)[53]. Therefore, the benefits and risks of psychotropic medications need to be considered, depending on the patients' symptoms and clinical judgment of the clinician[54].

Symptoms of major depression tend to overlap with what is considered to be normal changes in the postpartum period. For example, isolated occasional crying, weight changes, sleep disturbances, and fatigue are not uncommon among new mothers. Women lose weight after pregnancy and tend to consider sleep disturbances normal for the postpartum period. Frustration manifested by crying and fatigue further complicates the accurate assessment of PPD. Since women do not self-recognize their symptoms as abnormal, they are not likely to discuss them with health care providers. If screening does occur and women at high risk for PPD are referred to psychiatric care by their health care providers, women may not still attend appointments, often due to the stigma, sometimes due to lack of time and availability of convenient providers, and often because of symptoms themselves (lack of energy, motivation, and hopelessness). Minorities, such as African Americans, Hispanics, and other ethnic groups, are particularly at risk[55]. Language used by clinicians is important to consider in minimizing stigma. PPD may not be an acceptable term for mothers, especially for women from other cultures. A key challenge in diagnosis is the women's ability to self-recognize that they feel different and need treatment for PPD. Women may tend to normalize their symptoms and thus not discuss their symptoms with health care providers[56]. See Table 1 for a summary of symptoms, onset, impairment level, length of disorder, and diagnoses to rule out when assessing women for possible postpartum psychiatric disorders.

### *Screening*

The primary goal of the assessment process is to identify women at risk for PPD as well as women already in the depths of depression, and to evaluate women at immediate risk for self and others (e.g., suicidality, postpartum psychosis). Screening for PPD identifies more cases than relying on provider identification alone in a variety of patient populations and countries[57]. While the U.K. and Australia embraced routine screening, assessment for PPD has not become standard in the U.S. On a positive note, New Jersey enacted legislation that all licensed health care providers who provide perinatal care to screen for provide education and if needed resources for PPD[58]. Screening does not take the place of a complete psychiatric evaluation. However, early identification during pregnancy and the postpartum period would aid in referring women for further evaluation[50].

Self-report instruments that could be useful when screening for PPD include the Edinburgh Postnatal Depression Scale (EPDS)[59] and the Postpartum Depression Screening Scale (PDSS)[60]. The EPDS is a 10-question self-report instrument that rates symptoms on a scale of 0 to 3. A total score over 12 or 13 has been correlated with major depression with adequate sensitivity and specificity. This instrument has also been validated in numerous languages for assessing women from multiple cultures. The PDSS is a 35-item self-report instrument assessing seven symptoms on a scale of 1 (strongly disagree) to 5 (strongly agree). These symptoms include guilt/shame, mood changes, lack of concentration, anxiety, sleep/eating disturbances, loss of self, and suicidal ideation. Both instruments have excellent psychometrics, though the PDSS was found to have a higher sensitivity and specificity for major depression[61].

## Postpartum Psychosis

Postpartum psychosis is a severe, but rare, psychiatric disorder that may or may not coexist with PPD. In fact, only 1–4 in 1,000 women without prepregnancy or prenatal psychotic or bipolar episodes will experience postpartum psychosis, while about 4.5–9.3% of women may have postpartum psychosis if they had prepregnancy or prenatal psychotic disorders or bipolar episodes[62]. Women who have bipolar I disorder and family histories of postpartum psychosis are six times more likely to experience postpartum psychosis than women with bipolar I disorder alone[63]. Women diagnosed with postpartum psychosis are likely to experience further episodes of psychosis during their lifetime[64]. A large Swedish study found

**TABLE 1**
**Postpartum Psychiatric Disorders**

| | Baby Blues | Minor Depression | Major PPD | Postpartum Psychosis |
|---|---|---|---|---|
| Symptoms | Anxiety[2] about infant and parenting, teary, overwhelmed, fluctuation of positive and negative moods | Similar symptoms of major depression, but with less symptoms and less impairment[3] | Low mood, severe anxiety[1–3], feeling overwhelmed, possible panic attacks, hopelessness, suicidal thoughts[2] | Anxiety-vague, labile, low or elevated mood, preoccupied, distracted delusions and hallucinations |
| Onset | Within 10 days postpartum[3] | May start early postpartum | Within 4 weeks postpartum[3] | Acute onset, rapid, first few days to 2–3 weeks postpartum[2] |
| Level of impairment | Fluctuates, some good days, mood not necessarily low all day | Will be able to function, but with more effort | Feeling low most of the time for most days | Can deteriorate rapidly, psychiatric emergency |
| Time course | Usually improves over first few months | Lasts at least 2 weeks, all day | Incidence rises within 30 days[4], can last months, years[2] | Varies |
| Assessment | Follow to assess for minor depression/ major depression lasting more than 2 weeks [2] | Follow to assess worsening of symptoms to MDD, risk for suicide | Risk of suicide, neglect of infant and/or poor parenting, psychotic symptoms | May be harmful to self and/or infant due to poor judgment, command hallucination or delusional beliefs, needs hospitalization |

1 Chabrol, H. and Teissedre, F. (2004) Relation between Edinburgh Postnatal Depression Scale scores at 2-3 days and 4-6 weeks postpartum. *J. Reprod. Infant Psychol*. **22**, 33–39.

2 Buist, A. (2006) Perinatal depression-assessment and management. *Aust. Fam. Physician* **35(9),** 670–673.

3 American Psychiatric Association (2000) *Diagnostic and Statistical Manual of Mental Disorders 4th Edition: Text Revised*. American Psychiatric Association, Washington, D.C.

4 Cooper, P.J. and Murray, L. (1995) Course and recurrence of postnatal depression: evidence for the specificity of the diagnostic concept. *Br. J. Psychiatry* **166**, 191–195.

that women from poorer neighborhoods were 43% more likely to be admitted for postpartum psychosis than women from more affluent neighborhoods[65]. However, it is unclear how socioeconomic status influences onset of psychosis unless high-risk women are unable to continue employment. Family history of psychosis as well as bipolar I disorder appears to be a significant risk factor. The onset of postpartum psychosis tends to be rapid, occurring within the first 3–5 days after delivery and may last 1 week to several months without treatment. Symptoms include severe agitation, confusion, feeling of hopelessness and shame, insomnia, paranoia, delusions or hallucinations, hyperactivity, rapid speech, or mania. These delusions frequently focus on denying the baby's existence or the need to kill herself and/or the infant. This disorder requires immediate medical attention usually resulting in separation of the mother from the child, and psychiatric hospital admission. One pilot study found that the administration of 17β-estradiol diminished psychotic symptoms within 2 weeks in women with postpartum psychosis and estrogen deficiencies[66], suggesting a possible etiology of postpartum estrogen withdrawal in vulnerable women. There is limited research on postpartum psychosis treatments, especially preventative treatment in high-risk women.

## TREATMENT OPTIONS

### Nonpharmacological Treatments

Research has shown several types of psychotherapy to be effective for PPD: interpersonal psychotherapy (IPT), cognitive behavioral therapy (CBT), and group/family therapy. Two small randomized controlled trials (RCTs) found symptom reductions in women diagnosed with PPD through nurse-delivered CBT[67], maternal infant dyad group therapy (M-IGT), and individual IPT[68] after 8 weeks of treatment. No data analyses identified either M-IGT or IPT to be superior for treatment of depression. One larger RCT ($n$ = 120) found IPT effective in decreasing PPD symptoms as opposed to waiting-list condition[69]; however, when various methods of psychotherapy and control are compared in one RCT (n = 193), depressive symptoms reduce at 4.5 months, but without any significant difference between treatments (CBT, psychodynamic therapy, and nondirective counseling) Further, there were no lasting effects of depression reduction at 9, 18, or 60 months postpartum[70]. One study tested adaptation of IPT to group settings without a control group, with significant reduction of depressive symptoms[71]. These study samples failed to include various minorities (only Caucasian women); thus, the effectiveness is unknown for other groups. Psychotherapies may be helpful for breastfeeding mothers who are reluctant to use or continue antidepressants[72,73,74,75]. However, the time commitment of therapy may pose a burden for new mothers. One study found that high-risk women who preferred counseling treatment (controlling for antenatal depression and demographics) were six to eight times more at risk to have depressive symptoms at 3 and/or 6 months postpartum than women who did not prefer counseling[76].

### Antidepressant Treatment

One case-control study found that women diagnosed with PPD presented with significantly more anxiety symptoms, had less treatment response to antidepressant medication, and were more likely to be experiencing marked or severe depression than nonpostpartum women with depression[77]. Therefore, treatment with antidepressant therapy needs to be tailored specifically for PPD. Furthermore, three case studies reported the onset of mania after antidepressant therapy with postpartum women[78]. Thus, caution is recommended upon the initiation of antidepressant treatment in women[79]. Table 2 summarizes antidepressant treatment studies to date.

One double-blinded RCT ($n$ = 109) compared the effectiveness of sertaline and nortriptyline for remission of PPD after 8 weeks of treatment in a mixed racial sample. There was no significant difference between the two therapies; however, women who responded to sertaline or nortriptyline had 50% reduction in depressive symptoms by 2 and 4 weeks, respectively. The side effect profile reported for sertaline was lower than nortriptyline. Furthermore, breast milk levels of both sertaline and nortriptyline were undetectable in breastfeeding women[80].

One early RCT ($n$ = 87)[81] compared fluoxetine alone, fluoxetine in combination with one or six sessions of psychotherapy, and counseling only. No significant difference in effectiveness was found for any of the treatments, and all groups had decreased depressive symptoms. No breastfeeding women were included in the sample. More recently, fluoxetine was shown to have a wide range of drug levels in breast milk during 6 weeks of treatment for ten mother-infant dyads[82].

One single-blind study found that 60% of women responded to fluvoxamine titrated over 8 weeks. However, the small sample size and lack of breast milk data limits these findings[83].

**TABLE 2**
**Summary of Antidepressant Therapy for PPD**

| Sample (*n*) | Design | Measure | Treatment | Length of Trial | Comments | Ref. |
|---|---|---|---|---|---|---|
| 8 | Pre-experimental | HSD | Bupropion SR | 8 weeks | 80% sample decrease of 50% symptoms, 20% remission | [84] |
| 15 | Pre-experimental | HSD, Kellner symptom questionnaire | Venalfaxine immediate release, flexible dose | 8 weeks | 80% remission | [85] |
| 109 | Double-blind randomized comparative | HSD | SERT, NTP | 8 weeks | 46–48% sample with 50% reduction symptoms in both groups | [80] |
| 6 | Open label trial | HSD | FLOUXM titrated 50 mg to 150 mg/day, *M* = 142 mg (SD = 20) | 8 weeks | 66% responded with decreased symptoms on HAMD (over 17 to 2), no breast milk collected | [83] |
| 87 | RCT double blind | EPDS, HSD | FLOUXT and placebo with 1 or 6 counseling sessions | 12 weeks | Excluded breastfeeding women, no significant difference | [81] |

HSD: Hamilton Depression Rating Scale; EPDS: Edinburgh Postnatal Depression Scale; SERT: sertaline; NTP: nortriptyline; FLUOXT: fluoxetine; FLUOXM: fluoxamine.

## Alternative Therapies

Research studies are examining other options for PPD treatment. For example, omega 3 fatty acid has nutritional value to pregnant women and their infant, and lower serum levels of polyunsaturated fatty acids (PUFA) have been shown to be predictive of PPD[86]. Other studies found similar results; low DHA increased the risk of PPD in a large sample (*n* = 112)[87] and high DHA in mother's milk and seafood consumption predicted decreased PPD prevalence[88]. In an animal model, temporal lobes and frontal cortex were most affected by a DHA-depleted diet[89]. Studies on DHA supplementation for PPD found mixed results. One small randomized trial (*n* = 16) found about 50% decrease in PPD symptoms after 8 weeks of eicosapentaenoic acid (EPA) and docosahexaenoic acid (DHA) 0.5 mg/day, 1.4 g/day, or 2.8 g/day. No significant difference was found between any of the doses for depressive symptoms[90]. Other studies examined the risk of PPD, fish intake, fish/olive oil, and DHA; however, they did not have any positive results[91,92,93]. Many of the participants were not diagnosed with PPD and relied on self-report. Side effects were not found, but more longitudinal studies with placebo groups would assist in differentiating the effectiveness of this modality for PPD.

Studies examined *Hypericum perforatum* (St. John's wort) for major depression. Recent evidence review of RCTs found no significant difference between *H. perforatum* and placebo for major depression[94]. One randomized placebo-controlled study examined neurobehavioral changes in developing mice exposed to *Hypericum* (0.75 mg/g of food consumed daily, comparable to dosages in humans) vs. placebo at 2 weeks before conception and during pregnancy. Findings found no significant difference between the treatment and placebo group in motor coordination, vocalization, cognition, motivation, depressive and anxiety-like behaviors, and social play[95]. Another case study examined histological changes in rats after exposure of *Hypericum* (100 vs. 1000 mg/kg/day, comparable to human dosages) 2 weeks before conception, antenatal, and breastfeeding. This study found significant liver and renal damage with both dosages. The severity of the damage was related to the increased dosage strength[96]. No RCT studies examined the effects of *Hypericum* in pregnancy, breastfeeding, or PPD in humans. Two small longitudinal studies found limited infant adverse events during *Hypericum* exposure and breastfeeding[97,98]. The available data do not support the use of *Hypericum* in postpartum treatment, and caution should be exercised.

Bright light treatment produced antidepressant effects in an open trial ($n$ = 16) of antenatal depressed women[99]. One letter reviewed two case reports that demonstrated 75% reduction in symptoms from light treatment[100]. A recent RCT ($n$ = 10) showed women with antenatal depression and 10 weeks of 7000 lux light box had significant improvement (effect size 0.43, $p$ = 0.001) compared with women who received 500 lux or 5 weeks of 7000 lux[101]. Treatment risks are limited. No postpartum studies of light treatment have been conducted, to our knowledge.

### Electroconvlusive Therapy

Electroconvulsive therapy has been effective in major depression disorder and may also be effective with PPD. However, no studies to date systematically examined this treatment option with postpartum women. Even without studies, clinically, ECT should be considered in severe, suicidal PPD and in postpartum psychosis.

### Hormonal Treatment

Estrogen and progesterone decrease drastically after placental delivery, and a possible “endogenous gonadal-steroid withdrawal” has been hypothesized to contribute to mood changes. Ahokas et al.[102] found sublingual 17β-estradiol effective in reducing severe postpartum depressive symptoms by half within the first week in women with low baseline serum estradiol concentrations. This study included breastfeeding women, with no reports of diminished milk supply or infant feeding difficulties. No adverse events from estrogen treatment (e.g., endometrial hyperplasia, thromboembolism) were reported. One double-blind placebo-controlled study ($n$ = 61) of women who were diagnosed with PPD randomized to 200 mg 17β-estradiol transdermal patches for 6 months or no treatment[103]. This study found significant differences between the treatment and placebo group. Fifty percent of the treatment group rated themselves lower than the study’s identified threshold of 14 on the EPDS at 1 month and 80% of the women reported below the EPDS threshold at 3 months. The placebo group did improve in the depressive symptoms; however, their scores on average were not below the EPDS threshold for at least 4 months. Although these data suggest a more rapid improvement with estrogens, additional larger studies are necessary, with different dosages, and with consideration for safety, before estrogen may be considered an established treatment for PPD[104].

Progesterone enhances GABA in the central nervous system, producing anxiolytic and hypnotic effects; therefore, it may play a role in treating PPD with comorbid anxiety. Although one double-blinded RCT ($n$ = 100) found that depot norethisterone enanthate given within 48 hours after delivery increased the risk of developing PPD at 6 weeks postpartum and significantly suppressed endogenous 17β-estradiol vs. placebo ($p$ < 0.0001)[105]. Caution is recommended in using progestin in women at high risk for PPD[105].

### Treatment Summary

In summary, many women with PPD remain untreated, possibly from lack of information about treatment effectiveness and residual effects of antidepressants in nursing infants[79]. Serotonin-selective reuptake inhibitors (SSRIs) are considered the drug class of choice. However, all antidepressants cross into breast milk and milk-to-plasma ratios do not accurately predict safety to milk supplies. According to pooled data, SSRIs, and specifically sertraline and paroxetine, offer the best safety profile[106]. The benefits of taking antidepressants will have to be individually weighted against the risks of infant exposure to psychotropics. Benefits will probably outweigh the risks when moderate to severe symptoms, with significant functional deterioration or progressive worsening, are in place. Obviously artificial feeding alleviates concerns of infant psychotropic exposure.

The treatment of postpartum psychosis is beyond the scope of this article, but it is based on combinations of antidepressants, antipsychotics and, considering the association with bipolar disorder, sometimes, mood stabilizers. ECT is another safe and effective alternative. Emergency psychiatric hospitalization of the mother is one of the most difficult decisions the psychiatrist must make in postpartum psychiatric disorders.

### *Emergency Psychiatric Hospitalization*

No formal diagnostic criteria and guidelines for postpartum psychiatric disorders exist to assist psychiatrists with these critical diagnostic and treatment decisions[107]. Most of the literature discussed postpartum psychosis as needing immediate hospitalization and separation of the mother from the child[108]. Collateral information is absolutely necessary, and evaluation of the degree of family support available for mothers is essential for the adequate risk/benefit analysis of separating the mother from the infant and hospitalization. When women have severe PPD symptoms (e.g., lack of interest in the infant, excessive concern for infant's health, impaired functioning, suicidal or homicidal ideation, and especially intent or plan), the safety of emergency hospitalization outweighs the concerns of separating the mother and infant, familial difficulties, and stigma[109].

### *Immunological Considerations*

Studies are under way to identify unconsidered vulnerabilities and triggers for PPD that may provide new prognostic factors and identify novel therapeutic targets for PPD. Among them, the role that the immune system may play in the etiology of PPD is under consideration, in the context that imbalances in the immune system are associated with mood disorders in general[110,111]. The relationship between depression and the immune system might be particularly relevant for PPD, considering the postpartum rebound from the relative immunosuppression necessary for the mother to immunologically tolerate the embryo and the fetus, the physical trauma of birth, the exposure at birth of open wounds to bacteria from the urinary and gastrointestinal tract, and the effects of estrogen-progesterone deprivation postpartum on the immune system.

## CONCLUSIONS

Research studies continue to examine the etiologies and spectrum of postpartum psychiatric disorders. Distinguishing postpartum mood disorders, improving identification of PPD, and treating with effective therapies can decrease the poor outcomes associated with chronic and severe depression in postpartum women. This literature review identified mixed findings among various treatment options available. Antidepressant treatment is relatively safe and effective. Psychotherapy offers some hope, and may be a good indication as a combined treatment or as monotherapy in milder cases in women who breastfeed and are opposed to medications. Randomized control studies are badly needed for all PPD treatments. At times, the benefits of hospitalization, including involuntary hospitalization, when psychotic, suicidal, infanticidal, or failure to sustain daily needs of mother or child considerations are present, outweigh concerns related to separating the mother and child; developmental implications in the infant; and stigma, guilt, and shame in the mother. Additional research is necessary to uncover better prognostic factors as well as new treatments for PPD and related conditions.
